# Lumbar muscle involvement in the occurrence of osteoporotic vertebral fracture

**DOI:** 10.1016/j.redii.2023.100037

**Published:** 2024-02-01

**Authors:** Constance Lambeaux, Franck Lapègue, Hélio Fayolle, Yannick Degboe, Hélène Chiavassa-Gandois, Hubert Basselerie, Céline Goumarre, Romain Bilger, Nicolas Sans, Marie Faruch-Bilfeld

**Affiliations:** a Radiology department, hôpital Pierre-Paul-Riquet, CHU Toulouse Purpan, avenue du Professeur-Jean-Dausset, 31300 Toulouse, France; b Nuclear medicine department, hôpital Pierre-Paul-Riquet, CHU Toulouse Purpan, avenue du Professeur-Jean-Dausset, 31300 Toulouse, France; c Rheumatology department, hôpital Pierre-Paul-Riquet, CHU Toulouse Purpan, avenue du Professeur-Jean-Dausset, 31300 Toulouse, France

**Keywords:** Paraspinal muscle, Osteoporosis, Sarcopenia, Muscle atrophy, Muscle composition

## Abstract

**Objective:**

To determine if a lumbar musculature deficiency (paravertebral – PVM – and psoas – PM – muscles) is associated with a higher prevalence of vertebral fractures in osteoporotic patients.

**Methods:**

To constitute the fracture group, data were collected retrospectively from patients with one or more recent osteoporotic vertebral fractures between T10 and L5 such as non-injected computerized tomography (CT), dual-energy X-ray absorptiometry (DXA). A control group was made by matching the patients on age, bone mineral density measured by DXA and gender. We analyzed PM and PVM atrophy based on cross-sectional area (CSA) adjusted to the body area as well as fatty infiltration on a 3-level scale and the average muscle density in Hounsfield units (HU).

**Results:**

One hundred seventeen patients were included in each group. The fracture group had a lower PVM CSA than the control group (2197.92 ± 460.19 versus 2335.20 ± 394.42 mm^2^.m^−2^, respectively *p* = 0.015), but there was no significant difference in the PM (746.92 ± 197.89 versus 731.74 ± 215.53 mm^2^.m^−2^, respectively *p* = 0.575). The fracture group had a higher grade of fatty infiltration than the control group (PM: 1.3 ± 0.46 versus 1.07 ± 0.25, *p <* 0.001; PVM: 1.93 ± 0.5 versus 1.74 ± 0.5, *p* = 0.003) and a lower average muscle density (PM: 26.99 ± 12.83 versus 33.91 ± 8.12 HU, *p <* 0.001; PVM: 3.42 ± 21.06 versus 12.94 ± 18.88 HU, *p <* 0.001).

**Conclusion:**

This study shows an association between a lack of axial musculature and the occurrence of osteoporotic vertebral fractures. Preventive strengthening exercises could be proposed to osteoporotic patients.

## Highlights

• At equivalent bone mineral density, osteoporotic vertebral fractures are more frequent in patients who had deficits in their paravertebral and psoas muscles.
• Paravertebral and psoas muscle deficit is associated with occurrence of vertebral fracture in osteoporosis.
• Muscle atrophy and fatty infiltration should be evaluated in osteoporotic patients with vertebral fracture.

## Abbreviations and acronyms

BMD: Bone mineral density
BMI: Body mass index
CSA: Cross-sectional area
CT: Computerized tomography
DXA: Dual-energy X-ray
GE: General Electric
HU: Hounsfield Units
L: Lumbar
MRI: Magnetic resonance imaging
PM: Psoas muscle
PVM: Paravertebral muscle
PY: Pack-Years
RIS: Radiology Information System
ROI: Region of Interest
SD: Standard deviation
T: Thoracic

## 1 Introduction

Osteoporosis is a public health problem, affecting about 200 million women in the world and is associated with a high rate of morbidity and mortality [1], [2], [3]. Postmenopausal women are particularly impacted; about 50% of women over 50 are at risk for an osteoporotic fracture [4]. Due to aging of the population, its prevalence is likely to increase [5]. Osteoporosis is associated with high morbidity and mortality because of its complications. The most frequent complications are vertebral fractures, which are responsible for functional disability, low back pain, immobilization and kyphosis. Thus, osteoporosis has a significant financial burden [6,7].

The main risk factor for osteoporotic vertebral fractures is the severity of osteoporosis define as low bone mineral density (BMD) at peripheral and central sites [8]. The FRAX® score describes risk factors that need to be explored in clinical practice: gender, age, history of fracture (vertebral and non-vertebral), personal history of low-energy fracture, history of femoral fracture in parents, active smoking, glucocorticoids, rheumatoid arthritis, chronic alcoholism, and presence of secondary osteoporosis [9]. Other risk factors are related to bone microarchitecture, collagen characteristics and microdamage, vertebral size and shape, and to the risk of falling [10,11,8].

Recommendations insist on an osteoporosis assessment when a person presents a vertebral fracture in order to initiate osteoporosis drug treatment [12]. There is currently no consensus on whether or how to assess the axial musculature in the presence of vertebral fractures. However, the lumbar musculature has a major role in maintaining the spinal balance and it is known that osteoporosis and sarcopenia are associated [13,14].

We hypothesized that changes in biomechanical constraints related to an alteration of the lumbar musculature (paravertebral – PVM – and psoas – PM – muscles) could promote vertebral fractures. In this case, prophylactic measures such as physiotherapy, including exercises to improve the strength and quality of these muscles in people at risk, could be an important means of preventing vertebral fractures. The aim of our study was to determine if a lumbar musculature deficiency is associated with a higher prevalence of vertebral fracture in osteoporotic subjects.

## 2 Materials and methods

### 2.1 Type of study

This was a retrospective study involving computerized tomography (CT) data of patients with osteoporotic vertebral fracture compared to patients without vertebral fracture. This study was conduct in the Department of Radiology of Toulouse University Hospital (France) from March 2003 to December 2019. This study was approved by a research ethics committee. An information letter with a non-objection form was sent to all patients involved in this non-interventional study. The patient data were anonymized.

### 2.2 Study population

Patients were selected from our hospital's radiology information system (RIS). All patients with an imaging report containing the words “cementoplasty” and/or “vertebroplasty” were eligible. The inclusion criteria were patients who had one or more recent osteoporotic vertebral fractures between T10 and L5 visible on non-injected CT, a lumbar dual-energy X-ray absorptiometry (DXA) within 6 months of the CT scan and weight and height data available. The exclusion criteria were: non-osteoporotic vertebral fractures (pathological or traumatic), history of vertebral surgery, congenital spinal anomaly or scoliosis. To define a control group, we matched patients without vertebral fractures who had a non-injected CT covering at least T10 to L5 based on age (in 10-year increments), sex and BMD obtained by DXA (in 0.5 g/cm^2^ increments). Based on these criteria, we obtained two groups: patients with osteoporotic vertebral fracture (fracture group) and patients without fracture (control group).

Clinical data were collected from our hospital's computerized medical records database (Orbis), including substance abuse (tobacco and alcohol), chronic disease (diabetes, history of cancer or pathology requiring long-term corticosteroid treatment), previous or current specific osteoporosis therapy (bisphosphonate, teriparatide, calcium and vitamin D supplementation), risk factors for osteoporosis (early menopause, personal or family history of osteoporotic fracture, dysthyroidism), hormone replacement therapy for menopause [15,16].

### 2.3 Imaging protocol

The CT scans analyzed were lumbar spine, abdominal/pelvic or thoracic and abdominal/pelvic. They were acquired on two multidetector CT systems: Optima CT660HD or Discovery CT750HD (GE Healthcare, Waukesha, WI, USA). Acquisitions parameters were tube voltage 120 to 140 kV, tube current 200 to 370 mAs, slice thickness 1 or 1.25 mm.

DXA of the lumbar spine was performed using standard techniques on GE Healthcare Lunar (GE Healthcare, Waukesha, WI, USA).

### 2.4 Images analysis

#### 2.4.1 Vertebral fractures

Vertebral fracture was defined as an acquired focal or diffuse reduction in vertebral body height, diagnosed on CT or magnetic resonance imaging (MRI). The recent nature of vertebral fractures was determined by one of the following three criteria: the date of onset of pain, the absence of fracture on prior imaging (less than 1 months or the persistence of edema on MRI. We documented the level of vertebral fractures.

#### 2.4.2 Muscle analysis

Two muscle groups were analyzed: the bilateral PVM (multifidus and erector muscles) and PM. To set the reference section for all measurements, we chose the slice passing through the transverse processes of L3, parallel to the endplate [17], [18], [19], [20], [21]. We decided to study the L3 level because it is the most representative of overall body sarcopenia [20,18,17,19].

Measurements were taken on the CT scan performed prior to vertebroplasty as part of the preoperative assessment. Muscle analysis was performed by a radiologist with 5 years experience in musculoskeletal imaging.

Firstly, muscle atrophy was estimated by doing a free-hand drawing on Horos® software (https://horosproject.org/) to determine the region of interest (ROI) on the fascia of the four muscle groups of interest: PVM right and left, and PM right and left. Cross-sectional areas (CSA) expressed in square millimeters were adjusted to the patient's body area (Boyd's formula) (square millimeters per square meter), then both sides of the PVM and PM were added together to obtain PVM CSA (sum of PVM right and left) and PM CSA (sum of PM right and left) [22,23].

**Fig. 1:**
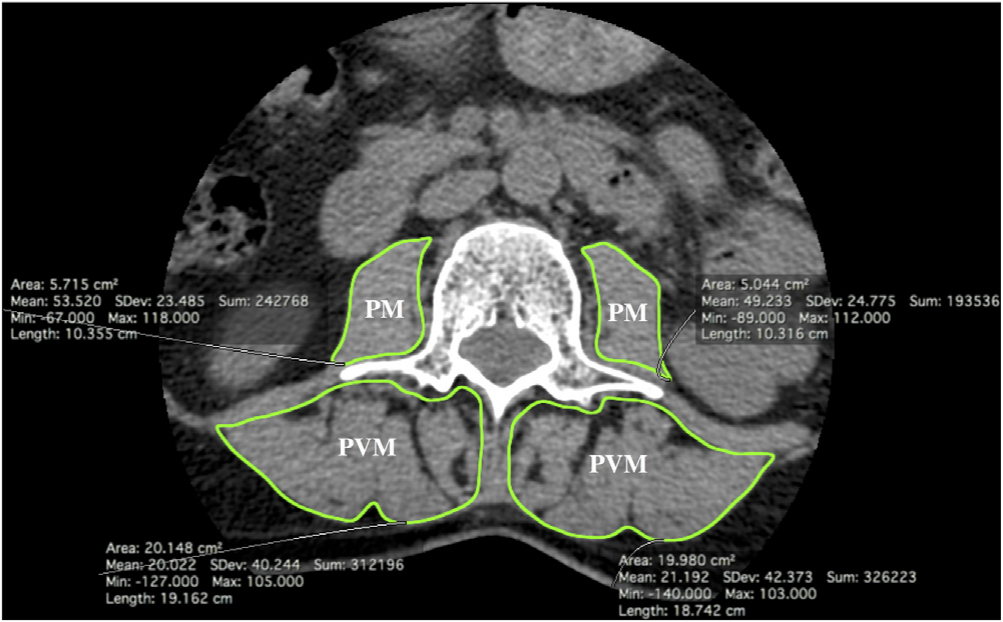
Muscular analysis using Horos software. Axial CT slice passing through the transverse processes of L3, parallel to the endplate. Muscles boundaries were drawn using a computer mouse.

Secondly, fatty infiltration was assessed in two ways: (i) average density in Hounsfield units (HU) was automatically calculated from the ROI, (ii) a semi-quantitative measure of visual fatty infiltration on a 3-level scale (mild grade 1 as a fat fraction below 10%, moderate grade 2 as 10–50% and severe grade 3 as above 50%) was made (Fig. 1) [18,20,24].

#### 2.5 Statistical analysis

Data were summarized as means and their interquartile range for quantitative variables and as counts with percentage for qualitative variables. Clinical characteristics of patients in the two groups were compared by a Chi^2^ or Fisher test, for qualitative variables, and Student's *t-*test or Wilcoxon-Mann-Whitney test for quantitative variables.

A univariate analysis, by Chi^2^ or Fisher test for qualitative variables, and Student's *t-*test or Wilcoxon-Mann-Whitney test for quantitative variables, was performed first.

A logistic regression was performed to look at the influence of muscle structure and atrophy on the occurrence of vertebral fracture.

A *p* value < 0.05 was considered statistically significant.

Statistical analyses were performed using R software (3.6) (https://www.r-project.org/).

## 3 Results

### 3.1 Population

**Fig. 2:**
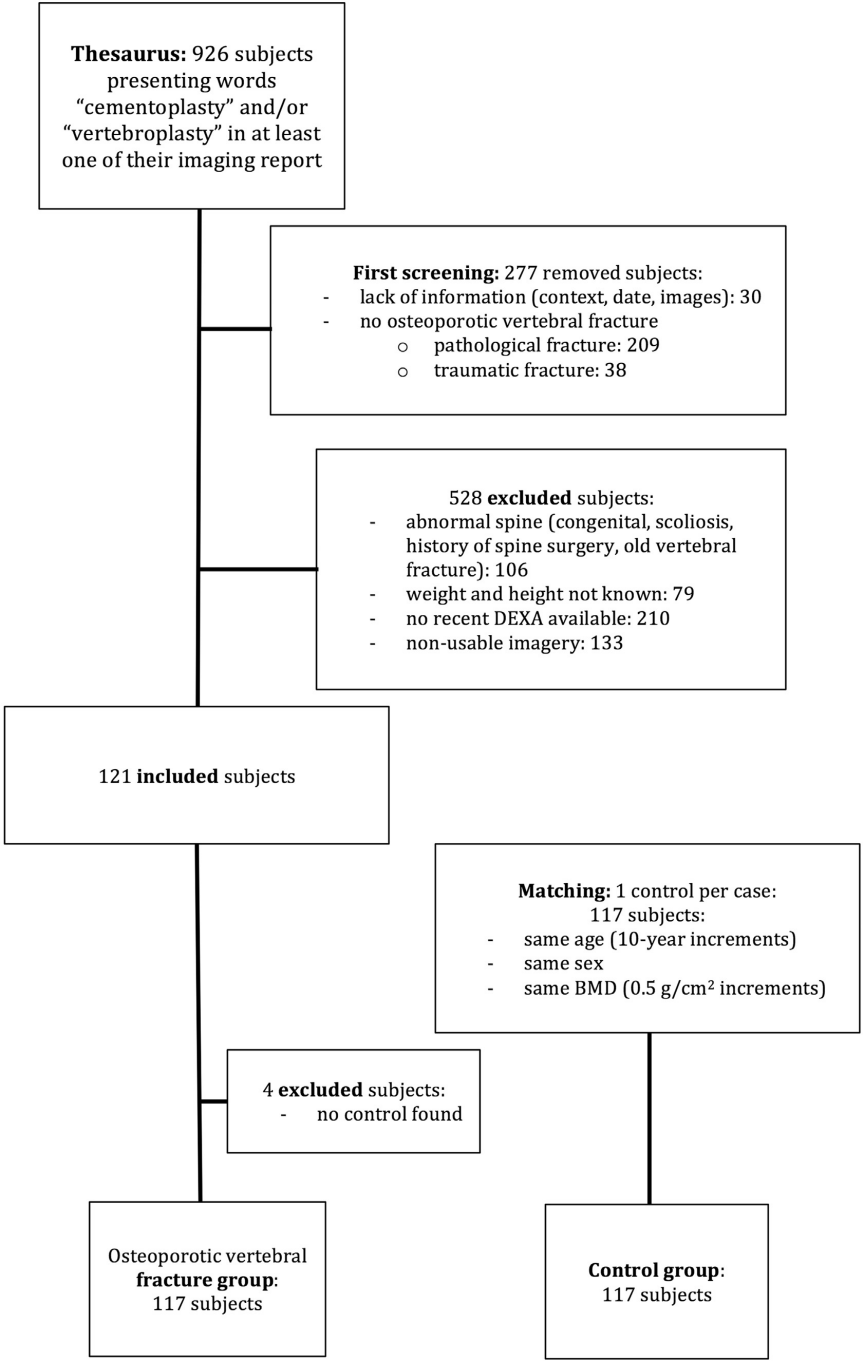
Patient selection flow chart.

A total of 117 subjects were included in the osteoporotic vertebral fracture group and 117 subjects were included in the control group (Fig. 2). An MRI showing edema of the fractured vertebra was available in 77% of patients in the fracture group, previous images less than one month old showing no fracture were available in 13% of cases, and 10% of patients in this group had a well-documented date of symptom onset of less than one month. The two groups were comparable in their age, gender, DXA value and body mass index (BMI) (Table 1) and body's area.

**Table 1:** Overall descriptive analysis.

	Overall	Fracture group	Control group	*p*-value
	*N* = 234	*N* = 117	*N* = 117	
		Mean or N (SD or%)	Mean or N (SD or%)	
Age (year)	68.3 (11.69)	68.22 (11.89)	68.38 (11.53)	0.920
Gender (female)	162 (69.2)	82 (69.2)	81 (69.2)	1.000
BMI (kg/m^2^)	26.47 (10.89)	26.68 (14.64)	26.25 (4.89)	0.765
Body area (m^2^)	1.76 (0.23)	1.74 (0.23)	1.78 (0.23)	0.15
BMD by DEXA (g/cm^2^)	0.94 (0.19)	0,93 (0.19)	0.96 (0.20)	0.296
Number of vertebral fracture		2.76 (1.88)	0	
Type 1 diabetes	7 (3.0)	3 (2.6)	4 (3.4)	0.497
Type 2 diabetes	36 (15.4)	15 (12.8)	21 (17.9)	0.497
Corticosteroids	106 (45.3)	40 (34.2)	66 (56.4)	**0.001**
Neoplasm	42 (17.9)	33 (28.2)	9. (7.7)	**<0.001**
Tobacco (PY)	9.56 (17.47)	12.09 (20.07)	7.03 (14.05)	**0.026**
Chronic alcoholism	26 (11.1)	17 (14.5)	9 (7.7)	0.145
Early menopause	32 (13.7)	24 (20.5)	8 (6.8)	**0.004**
Hypothyroidism	64 (27.4)	28 (23.9)	36 (30.8)	0.330
Hyperthyroidism	4 (1.7)	3 (2.6)	1 (0.9)	0.330
Menopausal hormone therapy	19 (8.1)	13 (11.1)	6 (5.1)	0.151
Personal history of fracture	61 (26.1)	33 (28.2)	28 (23.9)	0.551
Family history of fracture	9 (3.8)	6 (5.1)	3 (2.6)	0.497

BMI: body mass index; BMD: bone mineral density; PY: pack-year; SD: standard deviation.

**Fig. 3:**
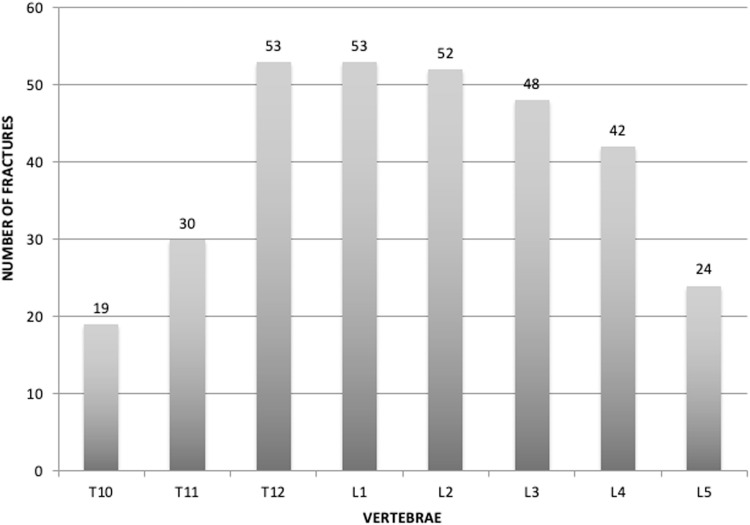
Distribution of vertebral fractures in the fracture group by level.

Patients in the fracture group had suffered an average of 2.76 vertebral fractures. The most frequently fractured levels were T12-L3. (Fig. 3)

Diabetes, chronic alcoholism, dysthyroidism, hormone replacement therapy, and personal or family history of fracture were not significantly different between the two groups. Corticosteroid use was significantly more frequent in the control group. In contrast, vitamin D supplementation, bisphosphonates and teriparatide were significantly more frequent in the fracture group. History of cancer, tobacco use and early menopause were significantly more common in the fracture group.

### 3.2 Muscle analysis

**Fig. 4:**
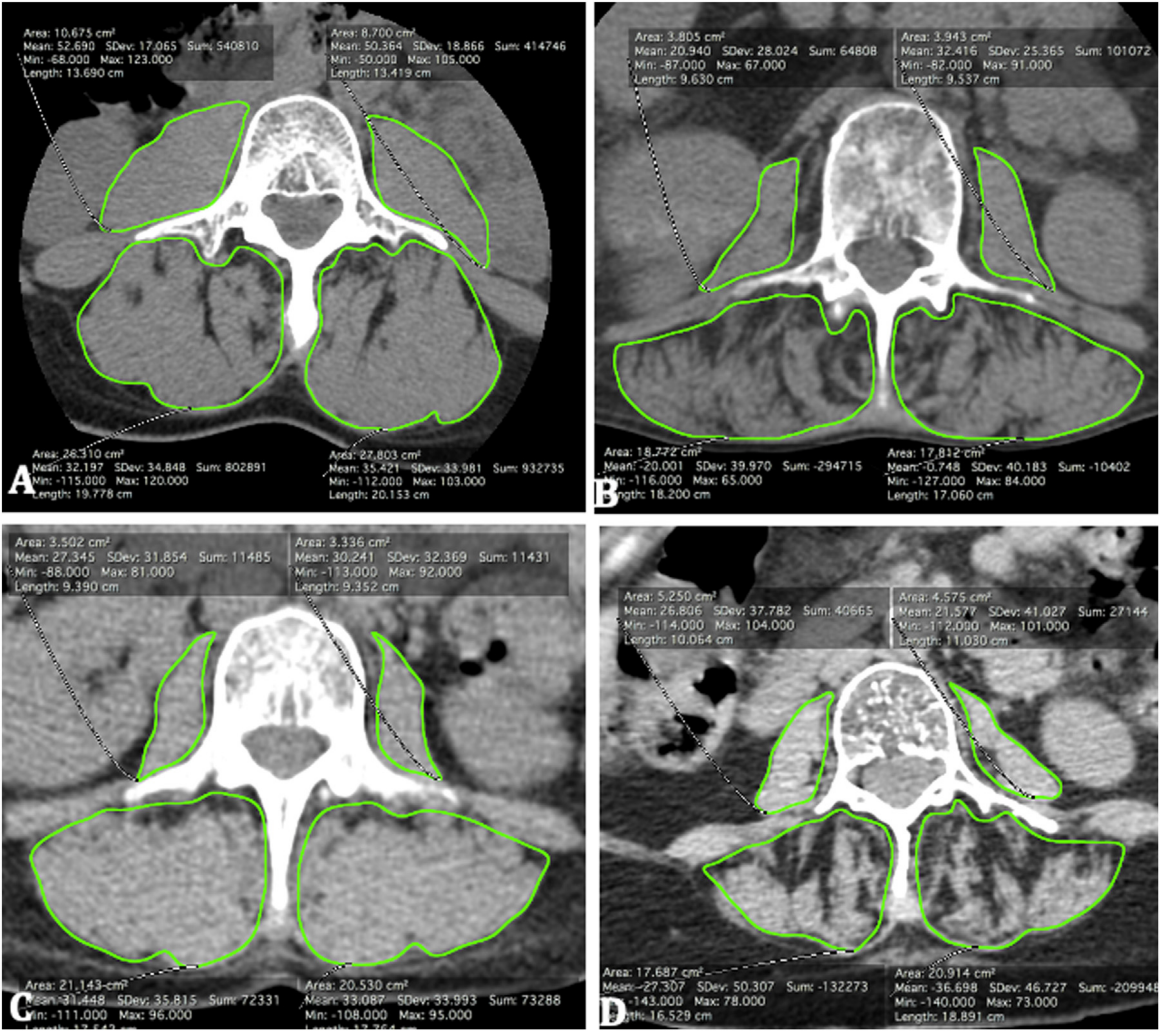
a-b Example of two male subjects, aged 40–50 years with bone mineral density on DXA between 0.5 and 1 g.cm^−2^. a Subject in the control group, CSA PM: 1196.9 mm^2^.m^−2^, CSA PVM: 3342.8 mm^2^.m^−2^. Average density of muscles: PM: 51.5 HU, PVM: 33.8 HU. Semi-quantitative fatty infiltration on 3-level scale: PM: 1, PVM: 1. b Subject in the fracture group, CSA PM: 530.1 mm^2^.m^−2^, CSA PVM: 2503.1 mm^2^.m^−2^. Average density of muscles: PM: 26.7 HU, PVM: −10.4 HU. Semi-quantitative fatty infiltration on 3-level scale: PM: 1, PVM: 2. c-d Example of two female subjects, aged 60–70 years with bone mineral density on DXA between 0.5 and 1 g.cm^−2^. c Subject in the control group, CSA PM: 462.3 mm^2^.m^−2^, CSA PVM: 2817.7 mm^2^.m^−2^. Average density of muscles: PM: 28.8 HU, PVM: 32.3 HU. Semi-quantitative fatty infiltration on 3-level scale: PM: 1, PVM: 1. d Subject in the fracture group, CSA PM: 624.8 mm^2^.m^−2^, CSA PVM: 2454.6 mm^2^.m^*−*2^. Average density of muscles: PM: 24.2 HU, PVM: −32.0 HU. Semi-quantitative fatty infiltration on 3-level scale: PM: 1, PVM: 3.

Muscle analysis Results from the muscle analysis are reported in Table 2 and Fig. 4.

**Table 2:** Comparison of muscle atrophy and fatty infiltration between the two groups.

			Overall	Fracture group	Control group	*p*-value
			*N* = 234	*N* = 117	*N* = 117	
				Mean or N (SD or%)	Mean or N (SD or%)	
Trophicity	Cross-sectional area (mm^2^/m^2^)	PM	739.33 (206.59)	746.92 (197.89)	731.74 (215.53)	0.575
		PVM	2266.56 (433.15)	2197.92 (460.19)	2335.20 (394.42)	**0.015**
Fatty infiltration	Density (HU)	PM	30.45 (11.26)	26.99 (12.83)	33.91 (8.12)	**<0.001**
		PVM	8.18 (20.52)	3.42 (21.06)	12.94 (18.88)	**<0.001**
	Semi-quantitative fatty infiltration	PM	1.18 (0.39)	1.30 (0.46)	1.07 (0.25)	**<0.001**
		PVM	1.83 (0.51)	1.93 (0.50)	1.74 (0.50)	**0.003**

HU: Hounsfield units; SD: standard deviation.

After normalizing to body area, the fracture group had significantly lower CSA for PVM, but not for PM than the control group (PVM: 2197.92 ± 460.19 versus 2335.20 ± 394.42 mm^2^.m^−2^, respectively *p* = 0.015; PM: 746.92 ± 197.89 versus 731.74 ± 215.53 mm^2^.m^−2^, respectively *p* = 0.575).

Average muscle density measured on CT was significantly lower in the fracture group for PVM and PM compared to the control group (PVM: 3.42 ± 21.06 versus 12.94 ± 18.88 HU, *p <* 0.001; PM: 26.99 ± 12.83 versus 33.91 ± 8.12 HU, *p <* 0.001).

**Fig. 5:**
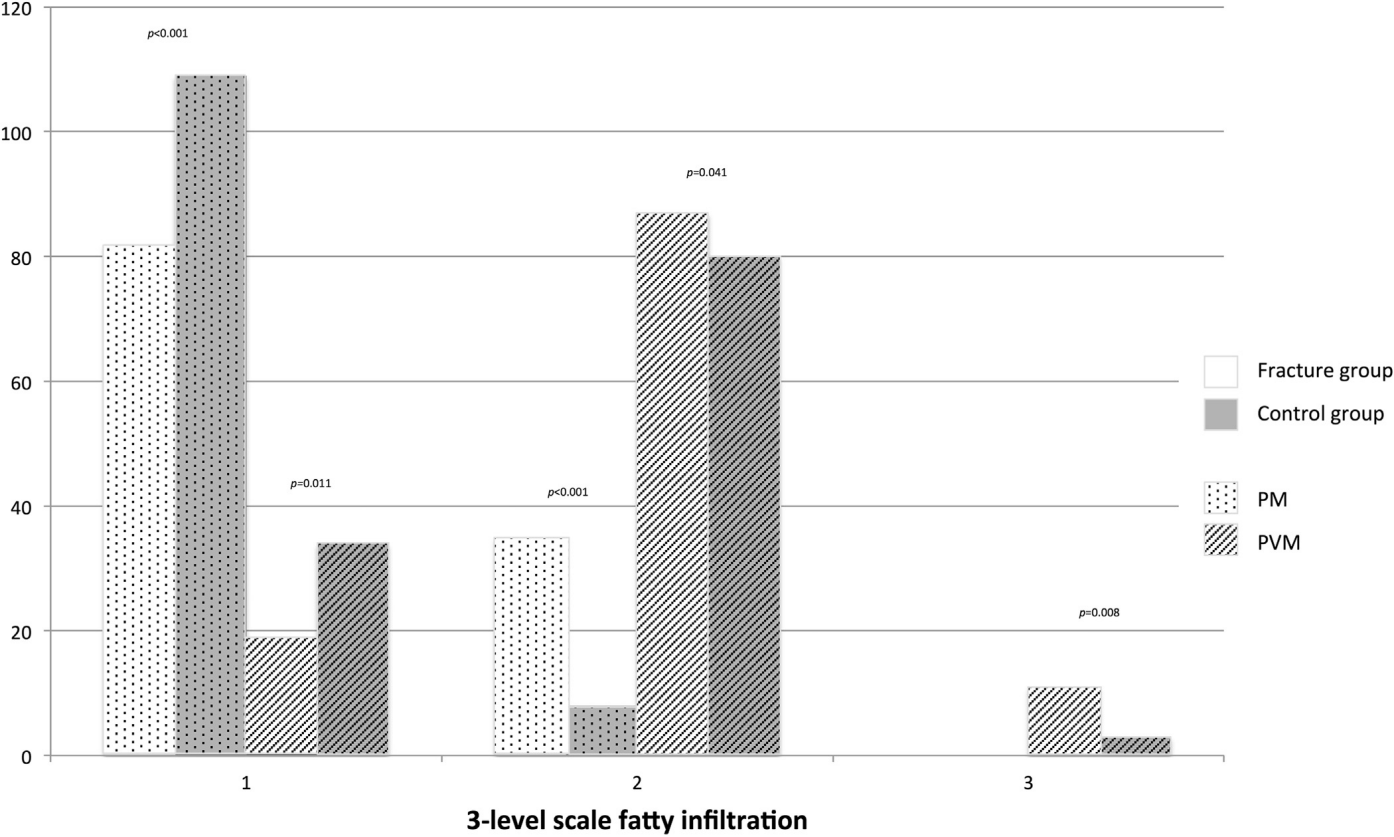
Histogram illustrating fat infiltration assessed semi-quantitatively on a 3-level scale (1: fat infiltration ≤10%; 2: fat infiltration between 10 and 50%; 3: fat infiltration >50%). PM: psoas muscles, PVM: paravertebral muscles. *P* values correspond to logistic regression.

Semi-quantitative analysis showed a higher fatty infiltration in the fracture group than the control group for PVM and PM (with a mean of 1.93 ± 0.5 versus 1.74 ± 0.5, *p* = 0.003 for PVM and 1.30 ± 0.46 versus 1.07 ± 0.25, *p <* 0.001 for PM). Fig. 5 displays the distribution of fatty infiltration grades by muscle group and whether a vertebral fracture is present or not.

## 4 Discussion

This study shows an association between the occurrence of vertebral fractures and deficient lumbar musculature. The role of lumbar musculature as a risk factor for vertebral fracture is not well known and unfortunately it is not part of routine clinical practice. According to Izzo et al., the axial musculature plays an essential role in active spinal stabilization [13]. According to Tzermiadianos et al., by participating in maintaining the static balance of the spine, the lumbar muscles limit the axial compression stresses that participate in generating vertebral fractures, particularly in osteoporotic subjects [25]. Huang et al. and Kim et al. also observed an association between osteoporotic vertebral fractures and lower paravertebral and psoas muscle volumes on MRI, but their cohorts were smaller (8 and 51 cases, respectively) and the subjects were not matched for BMD [26,24]. We chose to use CT data to have a larger sample of patients. To ensure that BMD was comparable between the two groups, we matched our patients on DXA data, the gold standard. In the fracture group, we also chose to use only examinations performed close to the fracture event because the presence of a fracture has an impact on the muscles and could be the consequence and not the cause of vertebral fractures [27].

The muscle CSA values cannot be compared between these studies because Huang et al. chose to estimate volumes from two slices while we used the cross-sectional area from only one section [26]. In addition, contrary to Huang et al. and Kim et al., we have chosen to relate the CSA of the muscles to the body surface area to normalize the results [26,24]. In contrast, compared with other studies that have investigated CSA of PM and PVM, our results are consistent, although not identical. In two studies on sarcopenia in a population of subjects with digestive cancers (but no information about vertebral fractures), the CSA values related to body surface area were comparable to those in our study [23,18]. Regarding muscle density, we found greater fatty infiltration than Dohzono et al. [18]. These differences can be explained in two ways: (i) it is possible that the measurements were taken on injected CT and (ii) the presence of a vertebral fracture was not specified in this population [18]. In our study, like in the one by Kim et al., PM fatty infiltration was mild (grade 1) in most patients in the control group, was more often moderate grade (grade 2) in the fracture group but was never severe (grade 3) [24]. In contrast, PVM fatty infiltration was most often moderate (grade 2) in both groups, but a greater proportion of the control group had mild fatty infiltration (grade 1), while severe fatty infiltration (grade 3) was more often found in the fracture group (Fig. 5). The lumbar muscles can be normal even in cases of osteoporotic vertebral fractures. On the contrary, a deficit in the lumbar musculature (for example: moderate or severe fatty infiltration) can be found even in the absence of osteoporotic vertebral fracture. Thus, it is an associated and predisposing factor for osteoporotic vertebral fractures, but not a determining one. Based on these retrospective studies, it is impossible to define a threshold value for the occurrence of vertebral fracture.

The difference in our study between the PM and PVM could be explained by their antagonistic role in maintaining stability. The PM is a flexor muscle, whereas the PVM (multifidus and erector spinae) are extensor muscles [13]. Since the mechanically stressed muscles in vertebra fractures are more anterior, the extensor muscles could have a more important role in combating these stresses (avoiding falls related to the kyphosis or the consequences of the kyphosis itself) [25].

In practice, the deficit in lumbar muscles is easy to study. Fracture exploration protocols include CT, which in our case provided a large cohort, and/or MRI. Although sagittal sequences were usually used, CUBE sequences, with the possibility of reconstructing axial sections, are increasingly being used. A quantitative evaluation of muscle atrophy is difficult to do in routine practice because it requires accurate contouring software and must be related to the body surface area. A semi-quantitative scale is easier to use in clinical practice. In our study, like in others, it is correlated with muscle density [28]. Moreover, in the coming years, chemical-shift-encoded water-fat MRI sequences (such as DIXON or IDEAL), will provide quantitative measurements of fatty infiltration with semi-automatic segmentation software [26].

Our study population in the fracture group was representative of the osteoporotic population. It included 68% women and 65% were over 65 years of age [29]. The distribution of fractures corresponded to that described in the literature. The most frequently fractured vertebrae were T12 and L1, followed by L2 and L3 [30].

The lumbar muscular deficit is an important parameter to study because there are safe, effective and accessible treatments to correct it for preventive purposes. Indeed, training at moderate and intense intensity helps to increase strength and muscle mass [31], especially since studies suggest a link between structural changes in the axial muscles and the risk of falling, which can lead to fractures [10].

Our study had several limitations. Interobserver reproducibility was not tested, but other studies have demonstrated acceptable reproducibility (according to Huang et al. Cronbach's alpha was 0.995 for muscle volume and 0.981 for intramuscular fat infiltration) [26]. Patients in the fracture group had history of cancer, tobacco use and early menopause, which could be confounding factors. In addition, they had been referred as part of a prevertebroplasty assessment, so it only included patients who were symptomatic about their fracture. The control group was made up of subjects being followed for other pathologies that required DXA and CT; this point is underlined by the higher prevalence of long-term corticosteroid therapy in the control group (56% in control group versus 34% in fracture group, *p* = 0.001) (Table 1).

## 5 Conclusion

PVM atrophy and fatty infiltration of the lumbar musculature (PM and PVM) is associated with the occurrence of vertebral fractures. Management by physiotherapy, including exercises to increase strength and quality of these muscles in the elderly, could be a relevant way to prevent vertebral fractures in at-risk subjects [31]. A supplementary prospective study would help to determine if structural modifications in these muscles is a risk factor for vertebral fracture.

## Disclosure

Constance Lambeaux, Franck Lapègue, Hélio Fayolle, Yannick Degboe, Hélène Chiavassa-Gandois, Hubert Basselerie, Céline Goumarre, Romain Bilger, Nicolas Sans and Marie Faruch-Bilfeld declare no relationship with any companies, whose products or services may be related to the subject matter of the article.

## Availability of data and materials

All data generated or analyzed during this study are included in this published article

## Ethics approval and consent to participate

Institutional review board approval was obtained (CRM-2105–164) and in accordance with retrospective review policies, formal consent was not required for this type of study.

An information letter with a non-objection form was sent to all patients involved in this non-interventional study.

## Declaration of generative AI and AI-assisted technologies in the writing process

During the preparation of this work the authors used DeepL Write in order to improve language and readability. After using this tool, the authors reviewed and edited the content as needed and take full responsibility for the content of the publication.

## CRediT authorship contribution statement

**Constance Lambeaux:** Conceptualization, Data curation, Investigation, Methodology, Writing – original draft. **Franck Lapègue:** Conceptualization. **Hélio Fayolle:** Formal analysis, Software. **Yannick Degboe:** Conceptualization, Methodology. **Hélène Chiavassa-Gandois:** Conceptualization. **Hubert Basselerie:** Conceptualization. **Céline Goumarre:** Conceptualization, Visualization. **Romain Bilger:** Conceptualization. **Nicolas Sans:** Conceptualization, Methodology, Visualization. **Marie Faruch-Bilfeld:** Conceptualization, Methodology, Supervision, Validation, Writing – review & editing.

## Declaration of competing interest

None.
